# Comorbidity of mental disorders with medical diseases in “Ali Mihali” Psychiatric Hospital, Vlora (2010-2020)

**DOI:** 10.1192/j.eurpsy.2022.1164

**Published:** 2022-09-01

**Authors:** E. Shaska

**Affiliations:** Ali Mihali” Psychiatric Hospital, Acute Service Unit, Vlore, Albania

**Keywords:** Mental Disorders, comorbidity, symptoms, medical diseases

## Abstract

**Introduction:**

The term comorbidity or dual diagnosis in this case refers to phsychiatric disorder and one or more medical diseases. The purpose of this study is to emphasize the importance of identifying medical diseases in psychiatry.

**Objectives:**

Medical diseases that develop most in comorbity with psychiatric disorders Identification of gravity of comorbidity symptoms Clinical progression Treatment efficacy/ Interaction of psychotropic medications with other medications.

**Methods:**

Methodology: A regular clinical study strategy has been adopted, with adults aged 19-75 females and males diagnosed with mental disorders and one or more medical pathologies, including neurological diseases during a 10-year period (2010-2020) in ”Ali Mihali” Psychiatric Hospital, Vlora.

**Results:**

They showed that medical diseases, such as: hypertension, diabetes mellitus, urinary infections, gastrointestinal disorders, acute and chronic bronchitis, severe brain injury often develop in comorbidity with mental disorders. Mental disorders that develop most in comorbity are: schizophrenia, schizoaffective disorder, delusional disorder, mood disorder. Treatment of these disorders is difficult due to the gravity of symptoms, interaction of medications, and side effects they have.

**Conclusions:**

Mental disorders in comorbidity with medical diseases are usually hard to treat. For this reason, it is imperative to diagnose them the soonest possible. When mental and medical disorders are comorbid, their coexistence has grave symptoms, chronic progression, which affects functioning, quality of life, and increases health care costs.

**Disclosure:**

No significant relationships.

